# Near fatal stent thrombosis in an aneurysmatic RCX as first manifestation of heparin induced thrombocytopenia (HIT) without thrombocytopenia

**DOI:** 10.1186/s12872-021-02442-3

**Published:** 2021-12-31

**Authors:** Marc Zoller, Iskandar Atmowihardjo, Jeanette Huch, Ines Albrecht, Dirk Habedank

**Affiliations:** 1 Intensive Care Unit, DRK Kliniken Berlin Köpenick, Berlin, Germany; 2 Medizinische Klinik Kardiologie, DRK Kliniken Berlin Köpenick, S.-Allende-Str. 2-8, Berlin, 12559 Germany; 3 Medizinische Klinik und Poliklinik B, University Hospital Greifswald, Greifswald, Germany

**Keywords:** Heparin-induced thrombocytopenia, HIT, Coronary aneurysm, White thrombus, Case report

## Abstract

**Background:**

Thrombosis resulting from heparin-induced thrombocytopenia (HIT) occurs in about 2% of patients without a significant decrease in platelet counts. We report on such a near fatal thrombotic event caused by coronary intervention.

**Case presentation:**

A supposedly “completely healthy” 53-year-old patient was admitted to hospital with covered rupture of an aneurysm of the Aorta descendens. He was successfully operated on and underwent coronary angiography due to NSTEMI six days later. Immediately after intervention of a 90% RCX stenosis he developed ventricular flutter, was defibrillated, and re-angiography showed partial occlusion of the RCX stent. Lots of white thrombi could be retrieved by aspiration catheter and gave reason for a HIT without thrombocytopenia. The detection of platelet factor 4/heparin complex antibodies by immunoassay supported and the subsequent Heparin Induced Platelet Activation Assay proved this diagnosis.

**Conclusions:**

The clinical event of an acute stent thrombosis should alarm the interventional team to the diagnosis of HIT even with a normal platelet count.

## Background

The incidence of heparin-induced thrombocytopenia (HIT) is approximately 1 in 5000 hospitalized patients [1], with a higher proportion of 0.5% after cardiac surgery [2]. Any new onset thrombocytopenia doubles the risk of early stent thrombosis after acute percutaneous coronary intervention (PCI) [3], even if HIT is confirmed in only 5–6% of patients with suspected HIT [4, 5]. In a still smaller proportion of about 2.2% of patients, thrombosis occurs without a significant decrease in platelet counts [5]. Against this background, we report on a patient with hyperacute and near fatal stent thrombosis as complication of HIT without thrombopenia.

## Case presentation

**Fig. 1:**
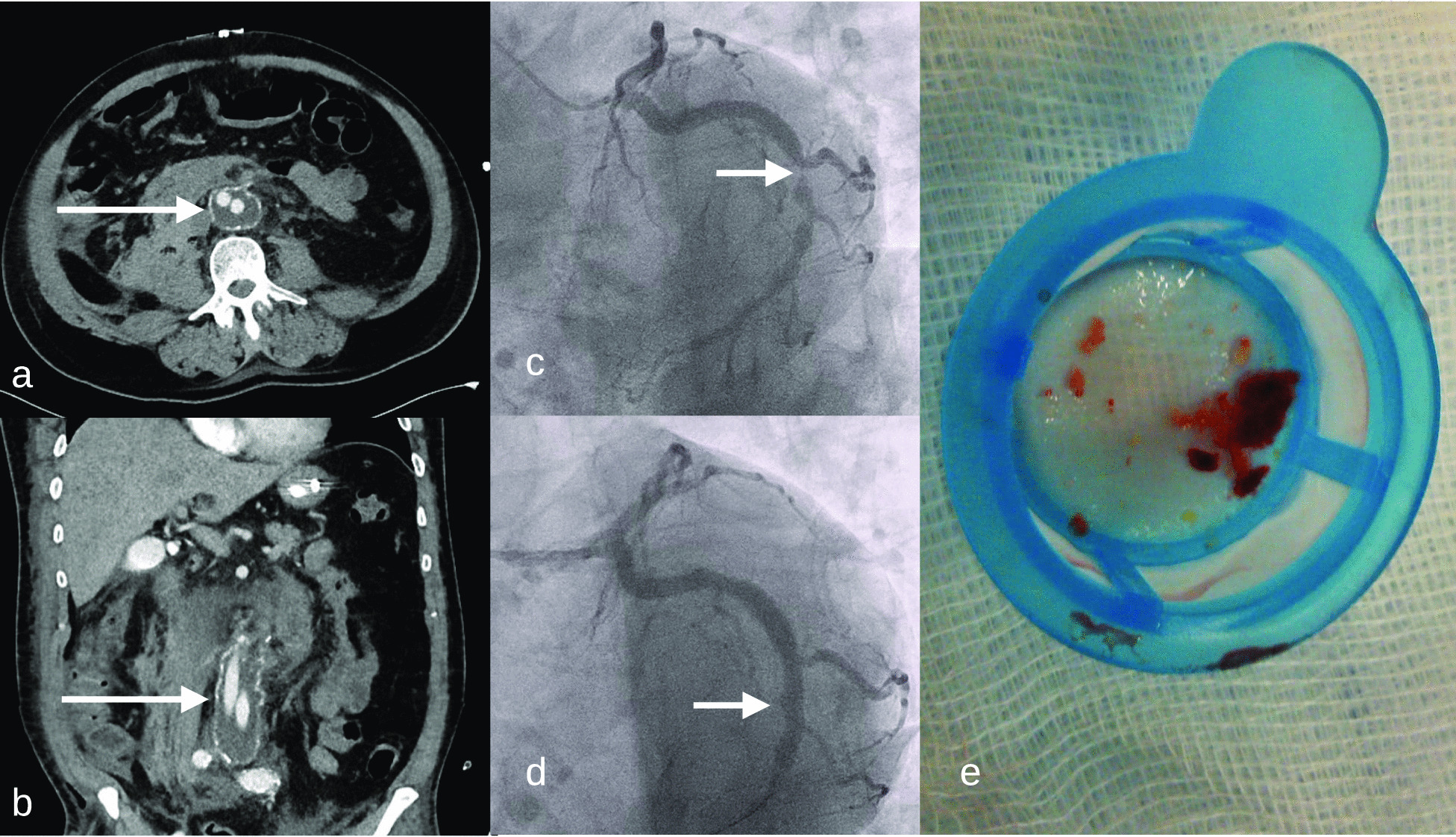
**a** Aortic aneurysma (long arrow) in CT axial view, **b** aortic aneurysm (long arrow) in CT coronal view, **c** coronary angiography showing aneurysmatic RCX (short arrow) before intervention, **d** coronary angiography with RCX after stent (short arrow) implantation, **e** the retrieved white thrombi

**Fig. 2:**
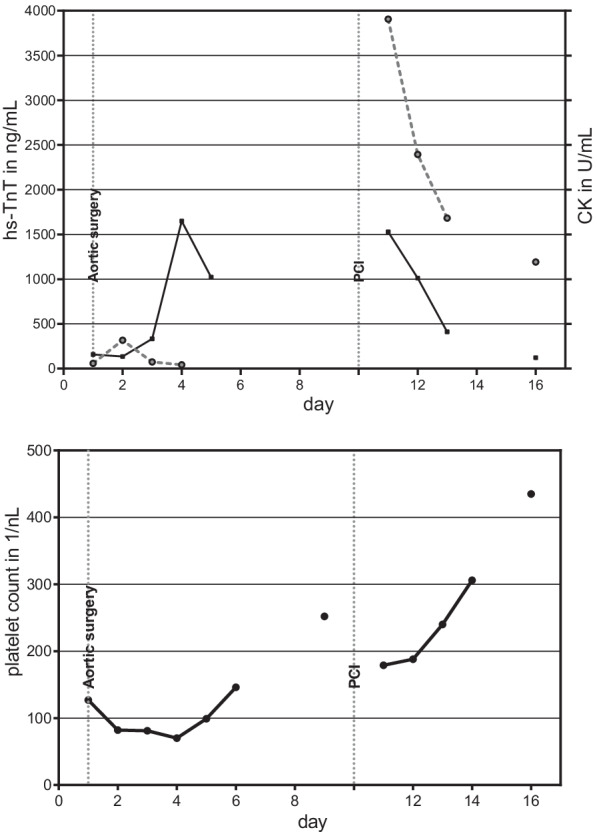
Cardiac laboratory markers and platelet count over time. Above: high sensitive troponin hs-TnT in ng/mL (circles and dotted lines), creatine kinase CK in units/mL (squares and line). Below: Platelet count in 1/nL (circles and lines), from admission (day 1) to discharge (day 16). Vertical lines indicate time course: Aortic surgery on day 1, PCI (percutaneous coronary intervention) on day 10

A supposedly “completely healthy” 53-year-old patient felt sudden annihilating abdominal pain and was admitted to our accidents and emergency unit. CT scans revealed a covered rupture of an aneurysm of the infrarenal Aorta descendens (Fig. 1a, b). He was immediately and successfully operated on and an aorto-bifemoral prothesis was implanted without further complications. The patient incurred a blood loss of about 1 L. In accordance with established guidelines the patient was administered 5000 IU of unfractionated heparin (UFH) prior surgery, which roughly equals a target of 60 IU/kg body weight. There had been no known hospitalization, surgery or other causes for heparin therapy for at least 12 months prior to admission. According to local ICU postoperative protocol the patient was started on 500 IU of UFH immediately for the five following days. He received a loading dose of 500 mg of aspirin after surgery and 100 mg daily thereafter. The platelet count had been 124 /nL on admission and decreased to a nadir of 70 /nL on day four before making a quick recovery (Fig. 2).

Initially elevated hs-troponin T decreased from 317 ng/mL (cut-off 50 ng/mL) to normal, ECG showed no signs of ischaemia, and the echocardiography proved a normal left ventricular function. Thus, coronary angiography was indicated but postponed to the end of the immediate postoperative phase. On day 6 on normal ward, i.e. 10 days after admission, coronary angiography was performed in physically and mentally stable condition, with normal platelet count (250 /nL, cut-off 150 /nL). The coronary angiogram showed aneurysms of the small RCA and the dominant RCX, thus confirming the generalized form of dilating atherosclerosis (Fig. 1c). The RCX aneurysm with interfered 90% stenosis was considered as culprit lesion. Thus heparin dosage for PCI was elevated from initially 5000 IU i.v. to 10.000 IU (100 IU/kg body weight) and the stenosis treated with a single drug eluting stent. Guiding catheter was already removed when the patient showed suddenly ST-elevations of 3 mV in leads II and III, remarkably without chest pain. The patient developed ventricular flutter, had to be defibrillated, and immediate re-angiography showed partial occlusion of the RCX. An aspiration catheter (Eliminate$\,^\mathrm{TM}$, Terumo Int. Syst.) retrieved lots of white thrombi (Fig. 1e), that have been linked to HIT events in vascular surgery in the past [6, 7]. Activated clotting time (ACT) at time of thrombus aspiration was 210 s (target range 240s). After intracoronary injection of eptifibatide 20 mg, the RCX had a TIMI-III-flow (Fig. 1d) and ST elevation decreased to 1 mV. In this emergency situation after defibrillation, re-opening of the complete vessel, TIMI-III-flow and with an uncertain prothrombotic situation we did neither perform an optical coherence tomography (OCT) nor a post-dilatation. The patient was transferred to the ICU. He remained free of angina pectoris and the ECG-elevation receded. Echocardiography remained unremarkable, and laboratory values showed a moderate increase in troponin (maximum 3900 ng/mL) and creatinkinase (1540 units/mL; Fig. 2). We administered acetylsalicylic acid 100 mg/d and ticagrelor 90 mg two times a day as platelet inhibition and fondaparinux 5mg s.c. as thrombosis prophylaxis. An immunoassay (Milenia Quick Line $^\mathrm{TM}$) detected platelet factor 4 (PF4)/heparin complex antibodies. Therefore a confirmation test had to be added and was immediately carried out as Heparin Induced Platelet Activation Assay (HIPAA, proprietary assay of Zentrum für Transfusionsmedizin und Zelltherapie Berlin gGmbH). The HIPAA returned positive and confirmed the diagnosis of HIT even in the absence of thrombocytopenia. The patient was transferred to normal ward and discharged from hospital 3 days later. In a telephone contact 6 months later, he had finished a rehabilitation programme and was in good condition.

## Discussion and conclusions

HIT is a prothrombotic and iatrogenic disease caused by antibodies to the platelet factor 4 (PF4)/heparin complex. It is characterized by thrombocytopenia and a high risk of limb- or lifethreatening thrombosis [5, 8]. Complex interactions between PF4, hematopoietic and endothelial cell surfaces limit thrombocytopenia while at the same time promoting prothrombotic processes. Thus, the main clinical manifestation of HIT is thrombosis, not bleeding [8]. The prevalence of heparin induced thrombopenia varies between 0.1 and 5%, depending on the type of heparin (UFH or low molecular weight heparin), the duration of exposure and the patient population [9]. It is also known that major surgery precipitates high concentrations of platelet factor 4 (PF4) and thus figures as an independent risk factor [10]. About 50% of patients with a HIT suffer thromboembolic complications. Among these large venous vessel thrombosis and pulmonary embolism are the most common, followed by embolisation of peripheral arteries and stroke. Myocardial infarction under HIT is thought to be rare under normal conditions [11, 12]. Other vessels like cerebral sinus or splanchnic veins have been known to be affected. White clots are indicative of a thrombocyte-related cause [6]. Typically, HIT develops in the interval from five to ten days after heparin exposure. A more acute form of the condition has been known as “rapid onset HIT” with sometimes only hours from exposure to life-threatening thrombosis, especially after prior exposure to heparin within the last 100 days [13]. A malicious situation is the incidence of HIT with normal platelet count. We assess that in our case any HIT-related drop in platelets was masked by a post-haemorrhagic thrombopoetic recovery. In a similar reported case this led to life threatening acute stent thrombosis that could not be solved by PCI and required immediate surgical revascularization [14]. A very recent case report by Mele et al. characterized this scenario as “easy to miss, uneasy to prevent” [15]. A HIT can be overlooked because acute PCI-related thrombosis is mostly related to morphological abnormalities. The French PESTO registry found mainly stent malapposition (48%) and stent underexpansion (26%) responsible for acute stent thrombosis [16]. OCT or intravascular ultrasound (IVUS) could rapidly prove this mechanical failure. As mentioned above, we did neither perform OCT nor IVUS in the acute situation, but we think that the long term positive outcome of our patient makes dissection or malapposition improbable.

In the case reported here several known risk factors combined made for a perfect storm. Not only had the patient received major emergency surgery on admission and a first pulse of UFH. Recovery from blood loss and coagulation factor depletion may have masked the clinical effects of antibody formation in the interval after surgery and allowed for platelet counts to recover up to normal levels. This kind of dynamic is not unheard of [1]. Moreover, HIT seropositivity does not inevitably lead to relevant pathology. Simultaneous high concentrations of heparin and PF4, as are common in vascular and cardiac surgery, are known to be associated with pathology [17]. Percutaneous coronary intervention requires therapeutic anticoagulation. In case of known HIT, bivalirudin or argatroban are substitutes of choice. We judge that the second (and elevated) UFH dose prior to PCI on day 6 was met with an primed immune response.

Tools in the diagnostic arsenal capable of uncovering disaster in the making are few. Anti-PF4-antibody screening tests are not available everywhere without significant delay. Moreover, the cost of testing renders it hardly viable as an indiscriminate screening method. Application of the “4T-score” (taking into account timing and severity of thrombocytopenia as well as the presence of thrombosis) helps to assess the probability of HIT as the underlying cause [18]. Up until the thrombotic event reported here the score would have returned a low probability. Without thrombocytopenia this score has to be considered 0, and after manifestation of the thrombotic event, the score would count 4 of a maximum 8 (2 points for the timing of thrombosis at day 6, and 2 points for acute and proven thrombosis). However, this score estimates the predictive probability of HIT in case of thrombocyto*penia* [13], and the positive predictive value is 10–20% for an intermediate score as in our case [19]. Detecting an evolving problem in its early stages remains crucial. The occurrence of thrombosis with no other established cause must trigger an immediate and decisive response. Or as it has been aptly put: “If you think HIT - act like HIT!”. Heparines and Danaparoid application should be stopped immediately and antithrombotic therapy continued using established alternatives. In a bailout situation like the one reported here, the additional use of GPIIb/IIIa-antagonists may be required.

**“Take-away” lesson:** HIT is a possible condition even with a normal platelet count. The clinical event of an acute stent thrombosis should draw the attention of the interventional team to this diagnosis.

## Data Availability

CT scans, angiography and laboratory values are available at the corresponding author by request.

## Abbreviations

ACT: Activated clotting time
CT: Computed tomography
HIT: Heparin-induced thrombocytopenia
ICU: Intensive are unit
IU: International units
NSTEMI: Non-ST-segment elevation myocardial infarction
OCT: Optical coherence tomography
PCI: Percutaneous coronary intervention
PF4: Platelet factor 4
RCA: Right coronary artery
RCX: Ramus circumflexus
UFH: Unfractionated heparin

## References

[1] Greinacher A (2015). Heparin-induced thrombocytopenia. N Engl J Med.

[2] Selleng S, Malowsky B, Strobel U, Wessel A, Ittermann T, Wollert H-G, Warkentin TE, Greinacher A (2010). Early-onset and persisting thrombocytopenia in post-cardiac surgery patients is rarely due to heparin-induced thrombocytopenia even, when antibody tests are positive. J Thromb Haemost.

[3] Oikonomou EK, Repanas TI, Papanastasiou C, Kokkinidis DG, Miligkos M, Feher A, Gupta D, Kampaktsis PN (2016). The effect of in-hospital acquired thrombocytopenia on the outcome of patients with acute coronary syndromes: A systematic review and meta-analysis. Thromb Res.

[4] Stoll F, Gödde M, Leo A, Katus HA, Müller OJ (2018). Characterization of hospitalized cardiovascular patients with suspected heparin-induced thrombocytopenia. Clin Cardiol.

[5] Greinacher A, Juhl D, Strobel U, Wessel A, Lubenow N, Selleng K, Eichler P, Warkentin TE (2007). Heparin-induced thrombocytopenia: a prospective study on the incidence, platelet-activating capacity and clinical significance of antiplatelet factor 4/heparin antibodies of the IgG, IgM, and IgA classes. J Thromb Haemost.

[6] Hermanns B, Janssens U, Handt S, Füzesi L (1998). Pathomorphological aspects of heparin-induced thrombocytopenia II (HIT-II syndrome). Virchows Arch.

[7] Okamoto H, Kume T, Fukuhara K, Kobayashi Y, Kawamura A, Goryo Y, Koyama T, Tamada T, Yamada R, Imai K, Neishi Y, Uemura S (2016). Optical coherence tomography findings in early stent thrombosis by heparin-induced thrombocytopenia. Int Heart J.

[8] Dai J, Madeeva D, Hayes V, Ahn HS, Tutwiler V, Arepally GM, Cines DB, Poncz M, Rauova L (2018). Dynamic intercellular redistribution of HIT antigen modulates heparin-induced thrombocytopenia. Blood.

[9] Martel N, Lee J, Wells PS (2005). Risk for heparin-induced thrombocytopenia with unfractionated and low-molecular-weight heparin thromboprophylaxis: a meta-analysis. Blood.

[10] Warkentin TE, Sheppard JA, Horsewood P, Simpson PJ, Moore JC, Kelton JG (2000). Impact of the patient population on the risk for heparin-induced thrombocytopenia. Blood.

[11] Warkentin TE, Kelton JG (1996). A 14-year study of heparin-induced thrombocytopenia. Am J Med.

[12] Almeqdadi M, Aoun J, Carrozza J (2018). Native coronary artery thrombosis in the setting of heparin-induced thrombocytopenia: a case report. Eur Heart J Case Rep.

[13] Warkentin TE, Kelton JG (2001). Temporal aspects of heparin-induced thrombocytopenia. N Engl J Med.

[14] Voudris V, Georgiadou P, Kalogris P, Kostelidou T, Karabinis A, Gerotziafas G (2019). Missed heparin-induced thrombocytopenia (HIT) diagnosis in a patient with acute stent thrombosis. Am J Case Rep.

[15] Mele M, Iacoviello M, Casavecchia G, Tricarico L, Brunetti ND (2020). Coronary thrombosis due to heparin-induced thrombocytopenia after percutaneous coronary intervention: easy to miss, uneasy to prevent. Clin Case Rep.

[16] Souteyrand G, Amabile N, Mangin L, Chabin X, Meneveau N, Cayla G, Vanzetto G, Barnay P, Trouillet C, Rioufol G, Rangé G, Teiger E, Delaunay R, Dubreuil O, Lhermusier T, Mulliez A, Levesque S, Belle L, Caussin C, Motreff P (2016). Mechanisms of stent thrombosis analysed by optical coherence tomography: insights from the national PESTO French registry. Eur Heart J.

[17] Rauova L, Zhai L, Anna Kowalska M, Arepally GM, Cines DB, Poncz M (2006). Role of platelet surface PF4 antigenic complexes in heparin-induced thrombocytopenia pathogenesis: diagnostic and therapeutic implications. Blood.

[18] Farner B, Kroll H, Kohlmann T, Warkentin TE, Eichler P, Greinacher A (2005). Clinical features of heparin-induced thrombocytopenia including risk factors for thrombosis. Thromb Haemost.

[19] Cuker A, Arepally G, Crowther MA, Rice L, Datko F, Hook K, Propert KJ, Kuter DJ, Ortel TL, Konkle BA, Cines DB (2010). The HIT expert probability (HEP) score: a novel pre-test probability model for heparin-induced thrombocytopenia based on broad expert opinion. J Thromb Haemost.

